# Dasatinib as a treatment for Duchenne muscular dystrophy

**DOI:** 10.1093/hmg/ddv469

**Published:** 2015-11-24

**Authors:** Leanne Lipscomb, Robert W. Piggott, Tracy Emmerson, Steve J. Winder

**Affiliations:** Department of Biomedical Science, University of Sheffield, Western Bank, SheffieldS10 2TN, UK

## Abstract

Identification of a systemically acting and universal small molecule therapy for Duchenne muscular dystrophy would be an enormous advance for this condition. Based on evidence gained from studies on mouse genetic models, we have identified tyrosine phosphorylation and degradation of β-dystroglycan as a key event in the aetiology of Duchenne muscular dystrophy. Thus, preventing tyrosine phosphorylation and degradation of β-dystroglycan presents itself as a potential therapeutic strategy. Using the dystrophic *sapje* zebrafish, we have investigated the use of tyrosine kinase and other inhibitors to treat the dystrophic symptoms in this model of Duchenne muscular dystrophy. Dasatinib, a potent and specific Src tyrosine kinase inhibitor, was found to decrease the levels of β-dystroglycan phosphorylation on tyrosine and to increase the relative levels of non-phosphorylated β-dystroglycan in *sapje* zebrafish. Furthermore, dasatinib treatment resulted in the improved physical appearance of the *sapje* zebrafish musculature and increased swimming ability as measured by both duration and distance of swimming of dasatinib-treated fish compared with control animals. These data suggest great promise for pharmacological agents that prevent the phosphorylation of β-dystroglycan on tyrosine and subsequent steps in the degradation pathway as therapeutic targets for the treatment of Duchenne muscular dystrophy.

## Introduction

The zebrafish *Danio rerio* has rapidly been adopted as an organism of choice for all aspects of the drug discovery pipeline (1–3). The zebrafish system offers unique advantages for drug screening in a vertebrate model organism, and in particular, muscular dystrophies are especially amenable due to their early, robust and readily recognizable phenotypes (4,5). The small size, embryonic status, low cost and ease of drug delivery directly via the water, makes zebrafish a very attractive model for whole-organism screening. Zebrafish show a typical vertebrate development pattern, and in the mutants, perturbation of muscle architecture and muscle function is readily observable even in the embryonic stages (4–6). In addition, of the genes known to be mutated in human forms of muscular dystrophy, many are represented in the zebrafish genome and those investigated so far exhibit dystrophic phenotypes in zebrafish (7,8). Although candidate compounds identified in fish would need to be validated in mammals before being taken on to human therapy, the low cost and speed of candidate drug screening, far outweigh any disadvantages.

Recent unbiased screens for DMD therapeutics have also validated this approach and identified a number of compounds that appear effective in reducing dystrophic symptoms in zebrafish (9,10). In particular, the identification of PDE5 inhibitors appears to be useful in this regard as they have also been shown to be effective in *mdx* mice (11,12).

Previous studies from the Lisanti group and ourselves suggested that tyrosine phosphorylation of dystroglycan is an important mechanism for controlling the association of dystroglycan with its cellular binding partners, dystrophin and utrophin, and also as a signal for degradation of dystroglycan (13–15). The Lisanti group further demonstrated that inhibition of the proteasome was able to restore other dystrophin glycoprotein complex (DGC) components in both *mdx* mice that lack dystrophin and in explants of DMD patients (16,17). As a first step, we examined the proteasomal inhibitor MG132 as a proof of principle in the zebrafish system comparing wild-type with dystrophic *sapje* larvae. As has been demonstrated for MG132 in mice and patient explants (16,17), we found that MG132 was also effective in *sapje* zebrafish in reducing the dystrophic phenotype (18). Moreover, in a genetic mouse model containing a tyrosine to phenylalanine mutation at residue 890 (Y890F) in β-dystroglycan, we demonstrated that preventing tyrosine phosphorylation of β-dystroglycan in *mdx* mouse alleviated the dystrophic phenotype (19). Taken together, these studies suggest a pathway in DMD where loss of dystrophin leads to increased phosphorylation of β-dystroglycan on tyrosine. This in turn results in the internalization and degradation of β-dystroglycan via the proteasome, leading to the loss of the entire DGC from the sarcolemma with an ensuing dystrophic phenotype. This pathway presents, therefore, three clear druggable targets through which to effect a treatment: inhibition of tyrosine phosphorylation of β-dystroglycan, inhibition of the ubiquitination of β-dystroglycan, and inhibition of the proteasomal degradation of β-dystroglycan. We have therefore tested candidate compounds with the relevant biological activities for their ability to reduce the dystrophic phenotype in *sapje* zebrafish and identified dasatinib as a potential therapeutic that could be repurposed to treat DMD.

## Results

**Figure 1.**
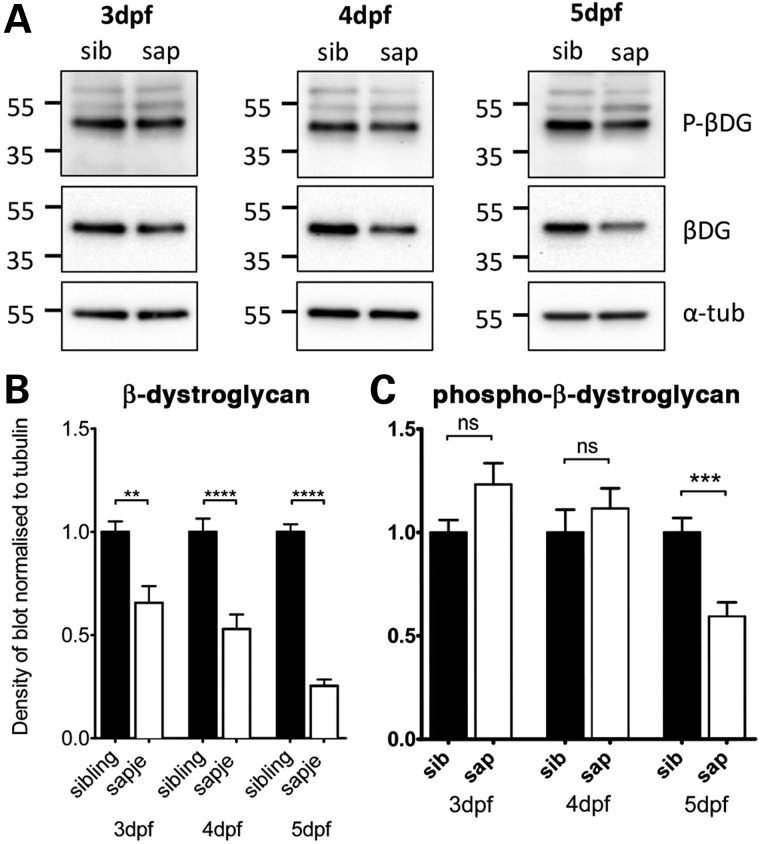
Levels of β-dystroglycan and β-dystroglycan phosphorylated on tyrosine in *sapje* and sibling larvae. Western blots of lysates of individual 3, 4 and 5 dpf sibling and *sapje* larvae western blotted with antibodies against phosphorylated β-dystroglycan [p-β-DG, (**A**) top], non-phosphorylated β-dystroglycan [β-DG, (A) middle] and α-tubulin was used as a loading control [α-tub, (A) bottom]. Numbers represent relative position of molecular weight markers in kDa. (**B** and **C**) The integrated density of the blots probed against β-DG and p-β-DG shown in (A), quantified relative to α-tubulin levels in each sample. Graphs show mean + SEM of 12 (B) or 9 (C) samples from three independent experiments in each case. (B) There is a significant decrease in the level of β-dystroglycan in larvae with the *sapje* mutation at 3, 4 and 5 dpf (unpaired *t*-tests, 3 dpf: *P* = 0.0016; 4 dpf: *P*<0.0001; 5 dpf *P* < 0.0001). (C) Levels of phosphorylated dystroglycan are slightly increased in *sapje* at 3 and 4 dpf, but this increase is not statistically significant (*P* > 0.05). When compared with sibling lysates, levels of phosphorylated dystroglycan at 5 dpf are significantly lower in *sapje* (*P* = 0.0007).

Homozygous *sapje* zebrafish show a progressive loss of muscle organization visible from 3 days post-fertilization (dpf) onwards (6,20). Concomitant with the loss of muscle organization, as observed by birefringence or fluorescence in whole embryos, is a progressive loss of immunoreactivity from the myosepta of other DGC components such as dystroglycan, compared with siblings (Supplementary Material, Fig. S1). The loss of other DGC components in the absence of dystrophin is common with other models of Duchenne muscular dystrophy (DMD) such as the *mdx* mouse (21), and in people with DMD (22). In order to more reliably quantify the extent of dystroglycan loss in *sapje* embryos, we performed quantitative western blotting of *sapje* and sibling larvae at 3, 4 and 5 dpf and examined the levels of β-dystroglycan, and β-dystroglycan phosphorylated on tyrosine, normalized to tubulin levels. As can be seen in Figure 1A, and in keeping with the immunofluorescence (IF) results in Supplementary Material, Figure S1, there is a progressive and significant loss of β-dystroglycan from 3 to 5 days in *sapje* larvae relative to siblings. In contrast to non-phosphorylated dystroglycan, tyrosine-phosphorylated β-dystroglycan does not decline until Day 5 (Fig. 1B and C). Therefore, there is a loss of non-phosphorylated β-dystroglycan that may contribute initially to the levels of tyrosine phosphorylated β-dystroglycan, but by 5 dpf, both non-phosphorylated and phosphorylated dystroglycan are significantly reduced. Also noticeable in Figure 1A, upper panels, is the appearance of higher molecular weight bands of tyrosine phosphorylated β-dystroglycan with a mass of ∼53 and 63 kDa, equivalent to 10 or 20 kDa heavier than the main 43 kDa β-dystroglycan band. Thus, the absence of dystrophin in *sapje* fish leads to a decrease in 43 kDa β-dystroglycan, with the concomitant appearance of slower migrating phosphorylated β-dystroglycan species. These data suggest a mechanism whereby in the absence of dystrophin, β-dystroglycan is more prone to phosphorylation on tyrosine; this results in a relative decrease in un-phosphorylated β-dystroglycan levels, and a concomitant increase in tyrosine-phosphorylated β-dystroglycan levels. This in turn leads to ubiquitin-mediated proteasomal degradation and a reduction in levels of all forms of dystroglycan by 5 dpf. The higher molecular weight dystroglycan bands may therefore represent ubiquitinated species. Assuming this is the case, the 10 and 20 kDa higher forms might represent the addition of one or two ubiquitin moieties.

**Figure 2.**
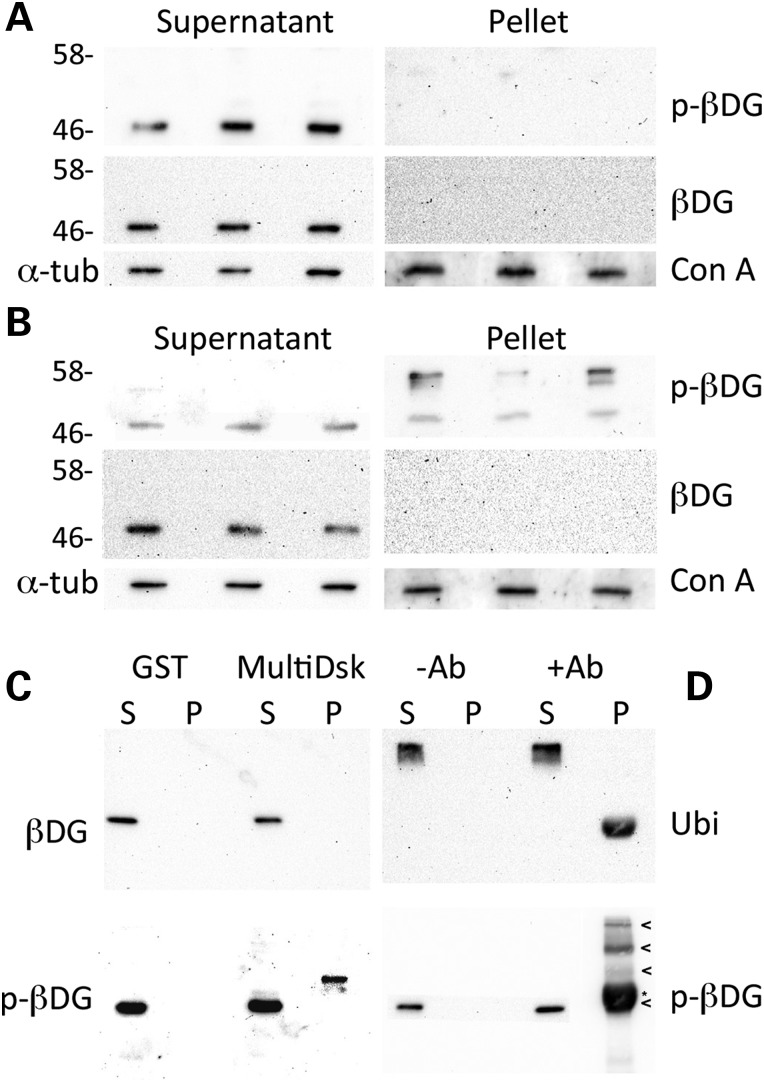
Phosphorylation and ubiquitination of β-dystroglycan in H2K myoblasts. Western blots for tyrosine-phosphorylated β-dystroglycan (p-β-DG, upper panels) or non-phosphorylated β-dystroglycan (β-DG, middle panels) from a pulse-chase surface biotinylation assay (19) at time 0 (**A**) and after 20 min of internalization (**B**). No dystroglycan is internalized to the pellet fraction at time 0 (A), whereas only tyrosine phosphorylated β-dystroglycan is recovered in the internalized pellet fraction at 20 min (B). Furthermore, note the appearance of higher molecular weight bands of tyrosine phosphorylated β-dystroglycan in the internalized fraction in (B). α-Tubulin (α-tub) and an unknown concanavalin A (Con A)-binding protein are used as loading controls for the supernatant (surface membrane fraction) and pellet (internalized fraction), respectively. (**C**) Supernatant (S) and pellet (P) samples from control GST and GST-MultiDsk pull-down were separated by SDS–PAGE and western blotted for pY890 β-dystroglycan [p-β-DG, (C) lower panel] or the unphosphorylated counterpart [β-DG, (C) upper panel]. Only tyrosine phosphorylated β-dystroglycan was pulled down by the MultiDsk ubiquitin binding protein. Immunoprecipitation from H2K myoblasts using p-β-dystroglycan antibody were western blotted probed for ubiquitin [Ubi, (**D**) upper panel] and phosphorylated β-DG [p-βDG, (D) lower panel]. Arrowheads in (D) indicate altered forms of p-β-dystroglycan, whereas the asterisk indicates non-specific immunoreactivity due to cross-reaction of rabbit IgG chains between the IP and blot. Numbers represent relative position of molecular weight markers in kDa.

We have previously used *H2k*b-tsA58 myoblasts to follow the fate of β-dystroglycan following tyrosine phosphorylation and internalization (19). Using a surface biotinylation and pulse chase approach, we were able to demonstrate that at 20 min following onset of endocytosis/labelling we were able to detect exclusively tyrosine phosphorylated β-dystroglycan in the internalized pellet fraction, whereas no internalized non-phosphorylated β-dystroglycan was detectable (Fig. 2A and B). Furthermore, the presence of two higher molecular weight bands was also evident, but again only in the internalized tyrosine-phosphorylated β-dystroglycan fraction (Fig. 2B). To identify the higher molecular weight, tyrosine phosphorylated β-dystroglycan bands, myoblast lysates were subject to pull-down with glutathione-*S*-transferase (GST)-MultiDsk (23) a protein comprising three repeated ubiquitin-binding domains fused to GST. As can be seen from Figure 2C, a higher molecular weight band was pulled down by the MultiDsk protein, but was only recognized by the antibody raised against tyrosine-phosphorylated β-dystroglycan and not by the antibody that detects unphosphorylated dystroglycan. These data suggest that internalized tyrosine phosphorylated β-dystroglycan is also ubiquitinated, and thus provides an explanation for the higher molecular weight bands in Figure 2B and as seen in whole zebrafish muscle in Figure 1A. Further confirmation of the ubiquitination of tyrosine phosphorylated β-dystroglycan was also provided by the reciprocal detection of higher molecular bands with an antibody raised against ubiquitin following pull-down of tyrosine phosphorylated β-dystroglycan (Fig. 2C).

**Figure 3.**
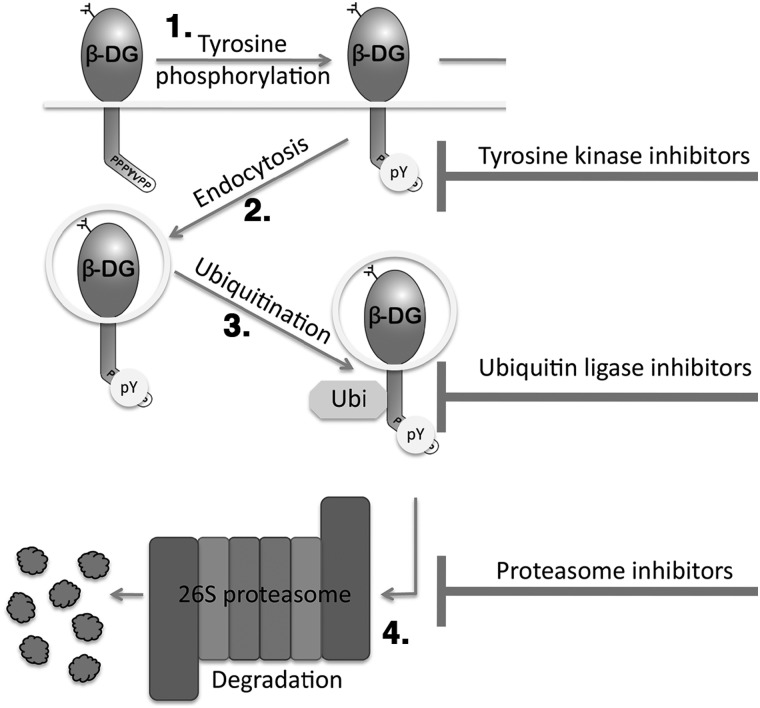
A schematic of the fate of internalized β-dystroglycan. β-DG is shown as a lollipop shape, inserted in membrane (represented by a line) with cytoplasmic domain directed down and the WW domain binding motif/Src tyrosine phosphorylation site PPPYVPP shown. Arrows show the sequence of events, with 1. Tyrosine phosphorylation by Src at Y890 (pY in circle) followed by 2. Endocytosis into a vesicle followed by 3. Ubiquitination (Ubi in octagon) and ultimately 4. Proteasomal degradation. T-shaped bars on the right indicate stages of the process that could be inhibited pharmacologically.

**Figure 4.**
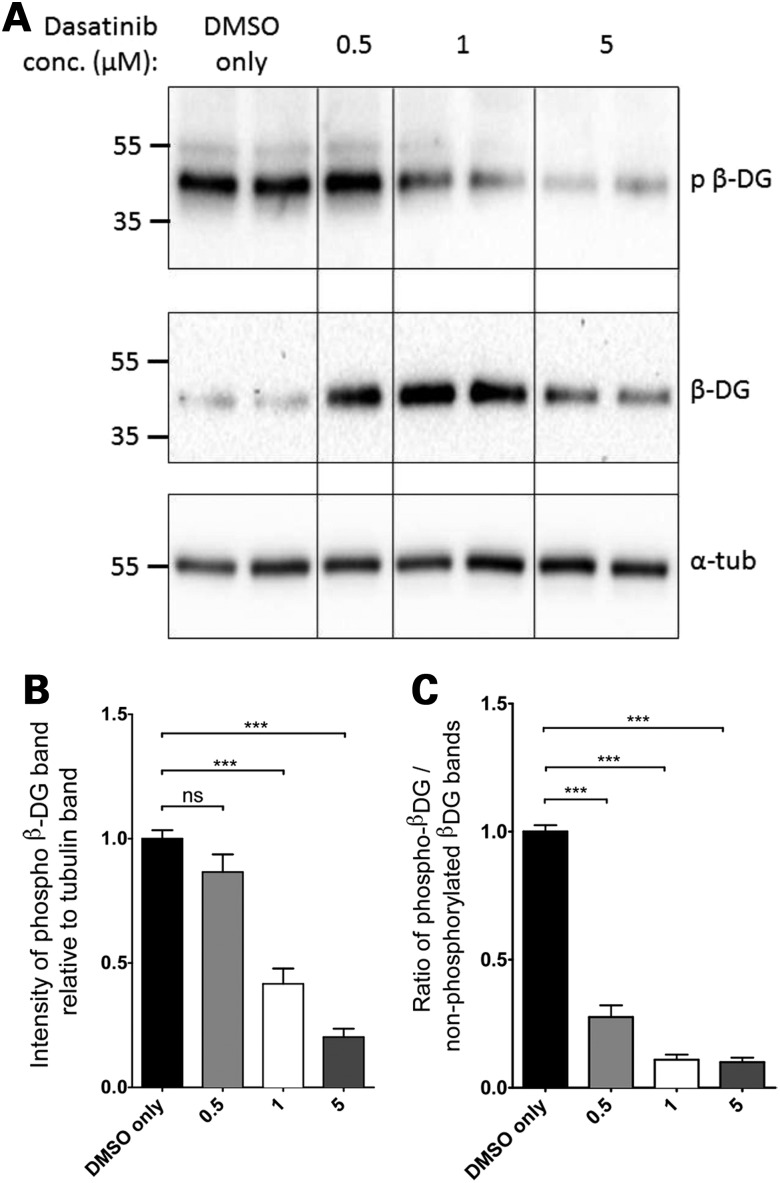
Phosphorylated β-dystroglycan in dasatinib-treated wild-type zebrafish larvae. Pools of 10 LWT zebrafish embryos were treated with dasatinib or DMSO from 24 hpf until 96 hpf and then lysed for SDS–PAGE. (**A**) A western blot of lysates probed with antibodies against phosphorylated β-dystroglycan (top panel), β-dystroglycan (middle panel) and α-tubulin (bottom panel). Numbers represent relative position of molecular weight markers in kDa. (**B**) The density of the blot probed against phosphorylated dystroglycan was quantified relative to α-tubulin levels in each sample, and represented as a ratio to the average DMSO only control signal. There is a significant decrease in the level of phosphorylated β-dystroglycan in dasatinib-treated embryos, compared with DMSO-treated controls. (**C**) The density of the blot probed against phosphorylated dystroglycan was quantified relative to non-phosphorylated dystroglycan levels in each sample, and normalized to the average DMSO only control signal. There is a significant decrease in the ratio of phosphorylated to non-phosphorylated dystroglycan in dasatinib-treated embryos, compared with DMSO-treated controls. Graphs show mean + SEM of at least eight samples for each treatment, from three independent experiments. One-way analysis of variance (ANOVA) was carried out followed by Dunnett's multiple comparison test (ns, non-significant; ***P < 0.001).

Taken together, the data in Figures 1 and 2 further substantiate a mechanism whereby in the absence of dystrophin, β-dystroglycan is phosphorylated on tyrosine, internalized and ubiquitinated, then degraded (Fig. 3). Based on this pathway, we undertook a candidate-based approach in zebrafish to identify compounds or drugs that could inhibit this pathway at different points, and that could therefore potentially alleviate the dystrophic symptoms by preventing dystroglycan phosphorylation and degradation. We have demonstrated previously in a mouse genetic model, that preventing tyrosine phosphorylation of β-dystroglycan ameliorates the *mdx* dystrophic phenotype (19). As the *in vitro* and *in vivo* data suggest that Src-mediated tyrosine phosphorylation of β-dystroglycan is the initial step in the degradative cascade, we sought to determine using small molecule inhibitors of Src, whether *in vivo* in fish there was any effect of dystroglycan phosphorylation on tyrosine in the dystrophic phenotype. Using the selective Src kinase inhibitor dasatinib (24), we examined whether dasatinib affected the levels of tyrosine phosphorylated β-dystroglycan in London wild-type (LWT) zebrafish embryos. As can be seen in Figure 4, compared with dimethyl sulfoxide (DMSO) vehicle alone, dasatinib at 1 and 5 µm caused a significant reduction in the levels of tyrosine phosphorylated β-dystroglycan, with a corresponding increase in levels of non-phosphorylated β-dystroglycan. Furthermore, at all concentrations of dasatinib tested between 0.5 and 5 µm there was a significant reduction in the ratio of phosphorylated to non-phosphorylated β-dystroglycan.

**Figure 5.**
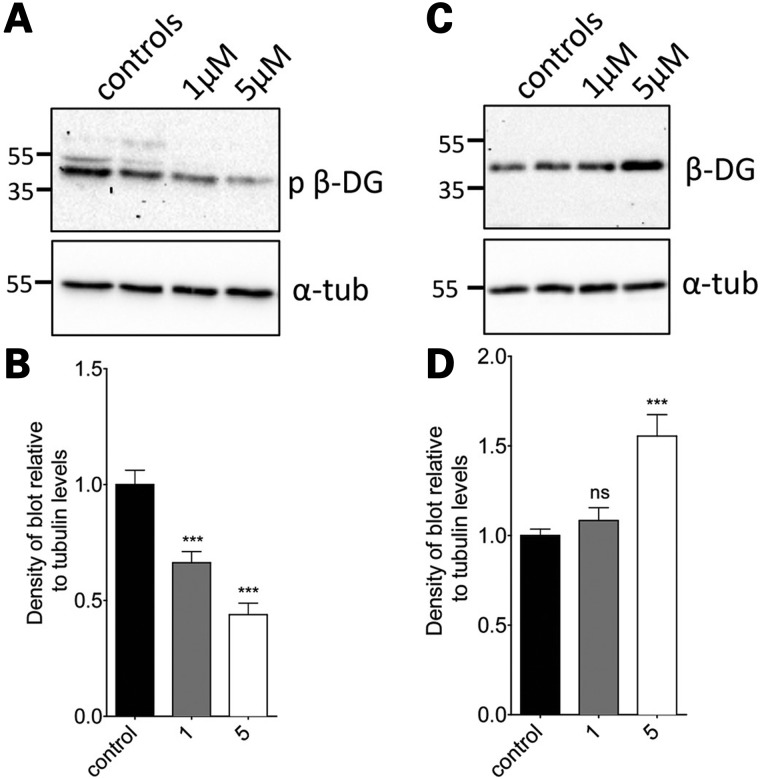
The effect of dasatinib treatment on levels of phosphorylated and non-phosphorylated β-dystroglycan in *sapje* zebrafish larvae. Lysates were made from *sapje*–/– embryos treated from 24 hpf until 96 hpf with dasatinib or DMSO-only. (**A**) Western blots probed with antibodies for p-β-DG and α-tubulin. (**B**) The density of the blot probed for p-β-DG was quantified relative to α-tubulin levels in each sample, and normalized to the average control signal. There is a significant decrease in the level of phosphorylated β-dystroglycan in larvae treated with dasatinib, compared with controls. (**C**) Western blots probed with antibodies for β-DG and α-tubulin. Numbers in (A and C) represent relative position of molecular weight markers in kDa. (**D**) The density of the blot probed against β-DG was quantified relative to α-tubulin levels in each sample, and normalized to the average control signal. There was a significant difference in the levels of β-DG in larvae between different treatment groups. Graphs show mean + SEM of at least six samples for each treatment, from three independent experiments. One-way ANOVA was carried out followed by Dunnett's multiple comparison test (ns, non-significant; ***P < 0.001).

**Figure 6.**
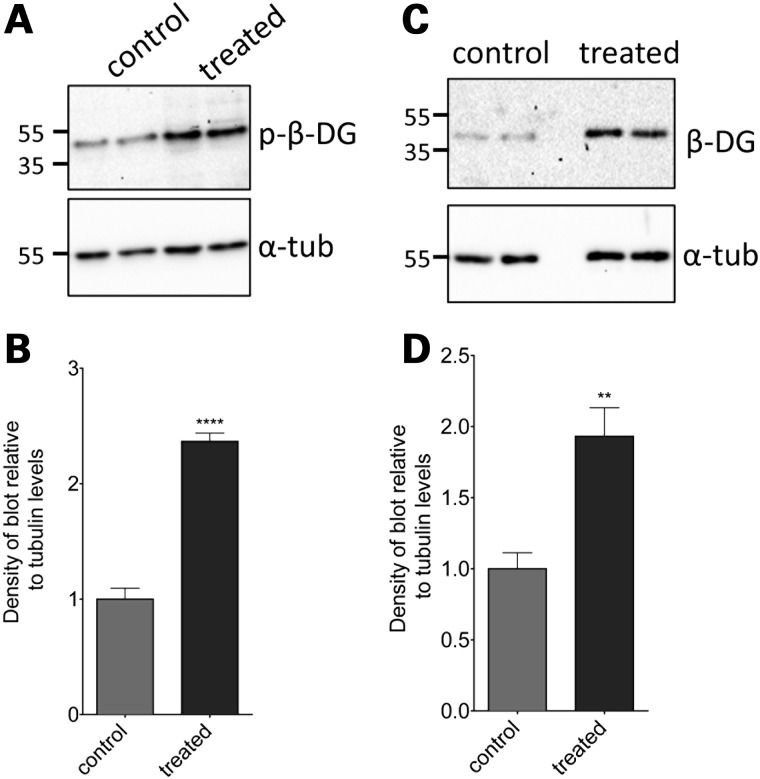
The effect of MG132 treatment on levels of phosphorylated and non-phosphorylated β-dystroglycan in *sapje* zebrafish larvae. Lysates were made from *sapje*−/− embryos treated from 24 hpf until 96 hpf with 5 μm MG132 or DMSO-only. (**A**) Western blots probed with antibodies for β-dystroglycan (β-DG) and α-tubulin (α-tub). (**B**) The density of the blot probed with β-DG was quantified relative to α-tubulin levels in each sample, and normalized to the average control signal. There was a significant increase in the level of β-dystroglycan in larvae treated with MG132, compared with controls (unpaired *t*-test: *t* = 4.048, df = 10, *P* = 0.0023). (**C**) Western blots probed with antibodies for phosphorylated β-dystroglycan (p-β-DG) and α-tubulin. (**D**) The density of the blot probed with p-β-DG was quantified relative to α-tubulin levels in each sample, and normalized to the average control signal. There was a significant increase in the levels of β-DG in larvae between different treatment groups (unpaired *t*-test: *t* = 11.47, df = 10, *P* < 0.0001). Graphs represent the mean of six samples from three independent experiments, error bars are SEM.

**Figure 7.**
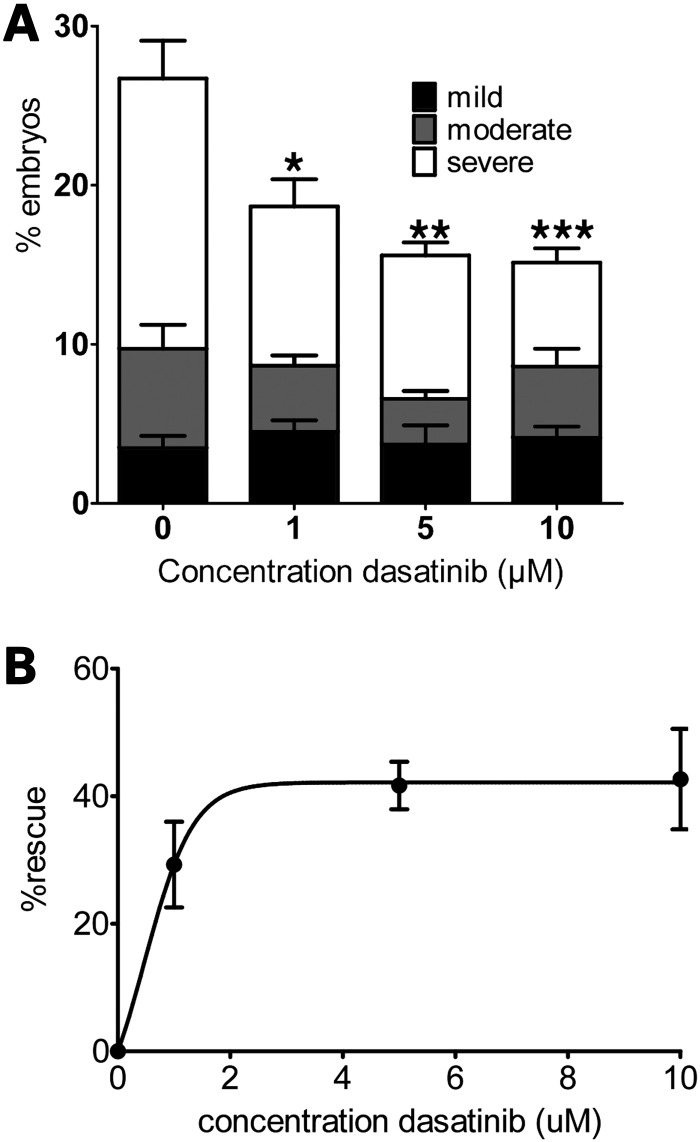
The effect of 72 h dasatinib treatment on *sapje* muscle phenotype. Embryos were treated with dasatinib or DMSO only for 72 h before a birefringence assay was carried out. The number of larvae showing normal or disrupted muscle birefringence was counted. (**A**) The proportion of larvae showing mild, moderate and severe muscle damage. There was a significant decrease in the percentage of larvae showing a severe phenotype in the groups treated with dasatinib compared with DMSO alone. (**B**) The overall percentage rescue of the dystrophic phenotype is plotted against dasatinib concentration. Data are plotted taking the proportion of dystrophic fish in DMSO-only treated groups as 0% rescue (one-way ANOVA followed by Dunnett's multiple comparison test: *P < 0.05, **P < 0.01 and ***P < 0.001). Data points represent mean values of five independent experiments, with a total of ∼250 embryos per treatment group. Error bars represent SEM.

**Figure 8.**
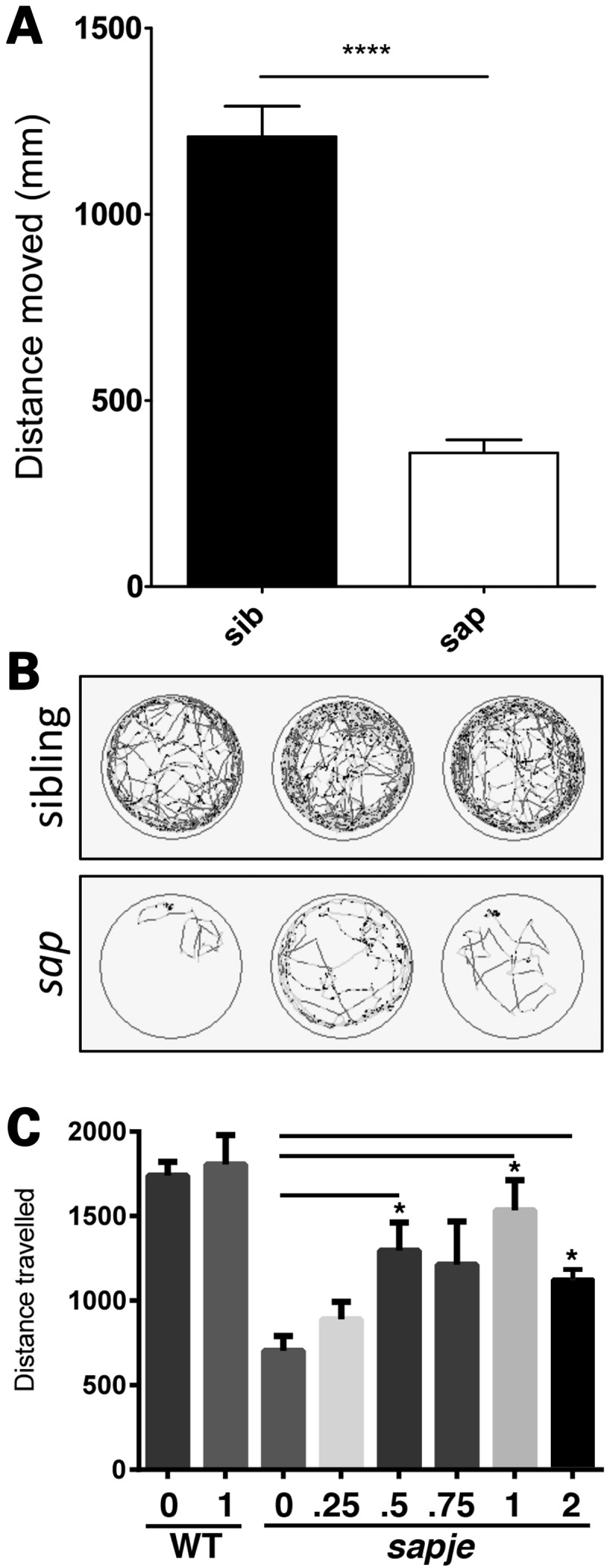
Dasatinib treatment rescues swimming ability in *sapje* larvae. Using ViewPoint video tracking and analysis, the overall distance moved by *sapje* larvae is significantly lower compared with wild-type siblings (**A**, *P* < 0.0001). Each bar represents mean tracking data from 36 larvae from three separate multiwell plates, and error bars represent SEM. (**B**) Representative traces for three *sapje* (*sap*, bottom) and three sibling larvae (top), light grey traces represent periods of fast movement and dark grey periods of slow movement. (**C**) *Sapje* and control larvae were treated with the indicated concentrations of dasatinib (µM), or DMSO only, from 3 to 5 dpf. Dasatinib has a dose-dependent effect in increasing swimming duration compared with vehicle-alone up to a maximum concentration of 1 µm. One micromolar dasatinib had no effect on swimming activity of wild-type zebrafish. One-way ANOVA analysis of ViewPoint tracking data indicated a significant increase between dasatinib-treated and control groups for the distance moved (one-way ANOVA: *F* = 3.188, df = 2.69, *P* = 0.0474, followed by Dunnett's multiple comparison test: *P < 0.05, ns = non-significant).

Having established that dasatinib treatment of normal zebrafish could alter the extent of dystroglycan phosphorylation, we next examined the effect of dasatinib in *sapje* zebrafish. As was seen in the LWT zebrafish, dasatinib at 1 and 5 µm caused a significant and dose-dependent decrease in β-dystroglycan phosphorylation, with a concomitant increase in the levels of non-phosphorylated β-dystroglycan (Fig. 5). Based on the scheme presented in Figure 3, the ultimate fate of phosphorylated dystroglycan is proteasomal degradation. We, therefore, investigated the effect of the proteasome inhibitor MG132 (25) on the levels of dystroglycan and phosphorylated dystroglycan in *sapje* zebrafish. As can be seen in Figure 6, MG132, like dasatinib, also increased the levels of both β-dystroglycan and phosphorylated β-dystroglycan, suggesting that blocking proteasomal degradation reduces the break-down of phosphorylated β-dystroglycan which accumulates in the muscle and further leads to increase in the levels of non-phosphorylated β-dystroglycan which is beneficial to the *sapje* fish. Quantitatively similar data were also obtained for the ubiquitin ligase inhibitor Pyr41 (26) (Supplementary Material, Fig. S2), where inhibition of the intermediate stages of the proposed pathway (outlined in Fig. 3) also resulted in a significant increase in the levels of both phosphorylated β-dystroglycan and non-phosphorylated β-dystroglycan. Therefore, inhibition of the tyrosine kinase resulted in an increase in the non-phosphorylated form of β-dystroglycan, and inhibition of the ubiquitin-proteasome pathway resulted in an increase in the levels of both the non-phosphorylated and phosphorylated β-dystroglycan. This is likely to be due to the phosphorylated dystroglycan not being degraded, which consequently leads to an increase in levels of non-phosphorylated dystroglycan. Furthermore, treatment of *sapje* fish with dasatinib from 24 to 72 hpf resulted in a significant and dose-dependent reduction in the proportion of embryos with a dystrophic phenotype (Fig. 7). The effect of dasatinib plateaued at ∼2 µm, with an estimated 40% of the dystrophic population displaying a normal muscle birefringence. When assessing the severity of the birefringence phenotype, there appeared to be a marked decrease in the percentage of fish showing a severe phenotype (Fig. 7A). While these data indicate that dasatinib can reduce the levels of dystroglycan phosphorylation, which from the mouse model (19) would be predicted to be beneficial, and produces an improvement in the integrity of the muscle as measured by birefringence (18), we also wanted to determine whether drug treatment had resulted in any improvement in muscle function. To test muscle function in the zebrafish embryos we used a high-speed video-tracking system to measure the swimming activity of individual fish over a 10 min period. When compared with normal siblings, it is immediately apparent from the tracks of *sapje* fish compared with normal siblings, that they are considerably poorer at swimming (Fig. 8A and B). Even when stimulated with convulsants such as pentylenetetrazole (27), *sapje* swimming ability was not increased (data not shown). All recorded measures of swimming activity were significantly lower in *sapje* fish including total time spent swimming, time spent swimming at fast or slow speeds and time spent swimming for short or long distances (data not shown), and total distance swum in 10 min (Fig. 8A). Dasatinib treatment of *sapje* fish between 3 and 5 dpf resulted in a significant increase in total distance swum in 10 min (Fig. 8C), indicating that the drug treatment not only has a biochemical and a histological effect, but also a physiological effect in increasing swimming activity in this fish model of DMD. Even at concentrations as high as 10 µm, however, dasatinib treatment had no effect on the birefringence phenotype of another dystrophic zebrafish model: the dystroglycan null zebrafish (Supplementary Material, Fig. S3). This strengthens the notion that the mechanism by which dasatinib treatment is able to improve muscle integrity is dependent on dystroglycan expression and its phosphorylation in the absence of dystrophin, as no improvement was seen in a dystrophic fish that lacks dystroglycan (Supplementary Material, Fig. S3).

## Discussion

The phosphorylation of β-dystroglycan on tyrosine acts as a molecular switch to regulate its interactions between different potential binding partners and different cellular adhesion functions (28). Phosphorylation of β-dystroglycan also acts as a switch to determine its intracellular fates, including internalization by endocytosis (15,19), trafficking to the nucleus (29–31) and, as we demonstrate here, proteasomal degradation. Dystroglycan phosphorylation was originally identified as a signal downstream of laminin engagement that led to regulation via alterations in the interactions between dystroglycan and several cytoskeletal ligands (14). The number of potential interactions and the degree of complexity of tyrosine phosphorylation regulated associations of dystroglycan have grown considerably in the last 10 years (28). Furthermore, with the identification of the kinase responsible—Src (32), the precise phosphorylation site (14,32), and with antibodies to distinguish the phosphorylated epitope, it has become clearer that tyrosine phosphorylation of dystroglycan is not simply a switch to modulate different binding partners during cell adhesion and migration, but also serves as a switch to control the internalization and intracellular trafficking of dystroglycan (15,19,33). Furthermore, tyrosine phosphorylation of dystroglycan serves as a specific signal for the endocytic uptake and proteasomal degradation of dystroglycan (19). We and others have hypothesized that this signalling event contributes to the aetiology of DMD due to its effect on the proteasomal degradation of not only dystroglycan itself, but also other components of the sarcolemmal DGC. Furthermore, Src kinase expression is elevated in *mdx*, the mouse model of DMD (34). As these analyses have revealed, tyrosine phosphorylation of dystroglycan appears to target the protein for endocytosis, resulting in ubiquitination leading to proteasomal degradation. This apparent pathway thus provides several points of potential therapeutic intervention, including inhibition of tyrosine kinases, preventing ubiquitination and blocking proteasomal activity. As previous analyses in zebrafish, mice, dogs and patient explants have shown, blocking degradative pathways pharmacologically with MG132 or bortezomib (Velcade) is able to restore components of the DGC to the sarcolemma, and has a beneficial effect on the dystrophic phenotype (16–18,35–37). However, one report of longer term treatment with the proteasome inhibitor MG132 failed to show any benefit (38). Bortezomib has also been demonstrated recently to benefit the dystrophic symptoms of the dy/dy mouse, a model of congenital muscular dystrophy MDC1A (39). Indeed, preventing phosphorylation and degradation of dystroglycan may be of benefit in other muscular dystrophies. For example, hypoglycosylation of dystroglycan in FKRP-deficient zebrafish invokes an unfolded protein response (UPR) (40) and increased degradation of dystroglycan. However, the role of tyrosine phosphorylation of dystroglycan in the UPR and subsequent degradation steps is still to be determined.

In our efforts to further dissect the signalling pathways responsible for DMD, and following the success of blocking phosphorylation of tyrosine 890 in dystroglycan in a mouse genetic model (19), we examined the effect of blocking tyrosine kinases on the zebrafish dystrophic phenotype. The Lisanti group had identified Src as the tyrosine kinase responsible for phosphorylation of dystroglycan at Y890 in a number of *in vitro* and cell culture assays (15,32). In our efforts to expedite the drug discovery pipeline for DMD, we therefore decided to examine Src inhibitors that were already FDA approved for use in humans, albeit for other indications, i.e. cancers. The drugs dasatinib, saracatinib and bosutinib all possess a low nanomolar IC_50_ for Src kinase (0.8, 2.7 and 1.2 nm, respectively) (41–43). These drugs have activity against the phosphorylation of dystroglycan on Y890 in mouse myoblasts and in wild-type and dystrophic zebrafish, and all improved the dystrophic phenotype in *sapje* fish (data not shown). Combinations of multiple kinase inhibitors, or kinase inhibitors with proteasome inhibitors, may lead to greater efficacy and the possibility of reduced side-effects. However, dasatinib was clearly the most effective leading to a 40% rescue of the dystrophic phenotype. While this is a significant improvement in dystrophic pathology, one has to question, given the hypothesis, why the extent of recovery is not greater. However, considering the 100% efficacy of the mouse genetic model in preventing phosphorylation of tyrosine 890, with only a partial amelioration of the dystrophic phenotype, it is perhaps not so surprising that the pharmacological treatment in zebrafish did not result in a full recovery. Moreover, these analyses are based only on our ability to monitor tyrosine phosphorylation at Y890, as this is the only tyrosine phosphorylation site that has so far been identified unequivocally (14,32), and our assumption that this phosphorylation event is solely responsible for the degradation of dystroglycan. In fact, our original analyses of adhesion-dependent tyrosine phosphorylation of dystroglycan revealed at least one other phospho-tyrosine containing spot on two-dimensional gels (14). More recently, it has been demonstrated that Lassa fever virus infection leads to tyrosine phosphorylation of dystroglycan, and importantly, although the level of pTyr was reduced when Y890 was mutated, it was not abolished, providing convincing further evidence for the existence of a second tyrosine phosphorylation site (44). Currently, we do not know which other tyrosine residues are phosphorylated, what contribution this has to the dystrophic phenotype, nor what kinase is responsible and hence whether dasatinib is effective at preventing this phosphorylation is unknown. With a disease like DMD, however, even small reductions in symptoms can have big impacts on quality of life. And while any one therapeutic approach is unlikely to give a 100% recovery, combinations of different therapeutic approaches aimed at stabilizing the DGC, for example, exon skipping to partially restore dystrophin expression, combined with tyrosine kinase inhibitors to reduce degradation of dystroglycan, could have an increased efficacy.

## Materials and Methods

### Zebrafish

Heterozygous *sapje^t222a^* (6), *dag1^hu3072^* (40) or LWT zebrafish were maintained as described previously (18). Animal experimentation was carried out in accordance with UK Home Office regulations and was approved by the local ethical committee. Embryos were collected and raised at 28°C under standard conditions (45). For all experiments, embryos were dechorionated at 24 hpf using 0.1 mg/ml pronase in E3 media for 30 min. Dechorionated embryos were then treated for various times with different concentrations of drugs or vehicle alone, as indicated in the results section. Drugs or vehicle (DMSO) was added directly to the E3 medium in multiwell plates at the start of the experiment and left for the duration without change. For treatments beginning before 3 dpf, embryos were treated in batches of 50 per well. The percentage of fish showing a muscle phenotype in each treatment group was compared with a DMSO-only control treatment group. DMSO-only treated fish were expected to show a muscle birefringence phenotype with Mendelian frequency of 25% *sapje* fish per group (actual recorded frequencies were 22–28%). For sodium dodecyl sulphate–polyacrylamide gel electrophoresis (SDS–PAGE) anaylsis, *sapje*–/– fish were selected post-treatment by birefringence. For embryos treated between 3 and 5 dpf, homozygote *sapje* embryos were sorted using birefringence prior to drug treatment. Embryos were treated as described above, but using 10 embryos per well. Phenotypically normal siblings were used as controls. The degree of rescue of the dystrophic phenotype was determined as described previously (18), and the severity of the muscle phenotype by counting the number of damaged somites. Fish were categorized as displaying wild-type, mild, moderate and severe phenotypes, shown in Supplementary Material, Figure S4c. Wild-type is undamaged, whereas mild, moderate and severe represent disrupted birefringence across 1–5, 5–10 or 10+ somites, respectively (Supplementary Material, Fig. S4). There were no survival or toxicity issues with any of the treatments used in the study. High-speed video-tracking motion analysis of zebrafish embryos was performed using a ViewPoint ZebraLab system (ViewPoint, Lyon, France) and as described in (27). To stimulate swimming activity, fish were subjected to four periods of 30 s light followed by 2 min dark over a 10 min period.

Immunofluorescence microscopy was performed on embryos fixed in 4% PFA at 4°C overnight or 2 h at room temperature. For labelling of F-actin, fixed embryos were incubated with 1:20 rhodamine phalloidin in phosphate-buffered saline (PBS) containing 0.1% Triton X-100. For antibody staining, fixed embryos were permeabilized with pre-cooled acetone at −20°C, blocked in PBS containing 1% bovine serum albumin, 1% DMSO and 1% Triton X-100, and incubated overnight in blocking solution containing the appropriate dilution of antibody. Imaging was carried out on an Olympus fluoview-1000 confocal microscope. For western blot analysis of zebrafish larvae, whole larvae were homogenized in RIPA buffer and boiled in SDS sample buffer before separation by SDS–PAGE and transfer to PVDF. *sapje*–/–larvae were identified by birefringence prior to homogenization (Figs. 5 and 6).

### Myoblasts

*H2k*b-tsA58 myoblast cells were maintained and subjected to surface biotinylation pulse-chase assay as described previously (19). The preparation and use of MultiDsk ubiquitin binding protein and use to pull-down ubiquitinated proteins has been described (23). Immunoprecipitation, SDS–PAGE and western blotting (WB) of β-dystroglycan was carried out as described in reference (46).

### Drugs, inhibitors and antibodies

Dasatinib was obtained from Selleckchem (Munich, Germany) and Pyr-41 and MG132 from Sigma-Aldrich (Gillingham, UK). The following antibodies were used in WB and/or immunofluorescence (IF) applications. Non-phosphorylated β-dystroglycan [MANDAG2; WB 1:100, IF 1:100 (47)], β-dystroglycan phosphorylated at tyrosine 892 [1709; WB 1:500 (13,19)], α-tubulin (WB 1:3500, Sigma-Aldrich), ubiquitin (WB 1:100, Enzo Life Sciences), biotinylated Concanavalin A (WB 1:4000, Vector Labs), western blots were detected by species-specific horseradish peroxidase conjugated to secondary antibodies or to streptavidin (1:5000, Sigma-Aldrich) and detected by ECL. In immunofluorescence, primary antibodies were detected by species-specific Alexa Fluor-conjugated secondary antibodies (IF 1:200, Molecular Probes) and F-actin was detected with rhodamine-conjugated phalloidin (IF 1:20, Molecular Probes).

## Supplementary Material

Supplementary Material is available at *HMG* online.

## Funding

The work presented in this paper was funded by a PhD studentship from the Muscular Dystrophy Campaign grant number RA/806 (L.L.) a PhD studentship from the MRC grant numbers G0900203-1/1 G1000405-1/1 (R.W.P.) and a project grant from the Duchenne Parent Project NL (S.J.W.). Funding to pay the Open Access publication charges for this article was provided by MRC (UK).

## Supplementary Material

Supplementary Data
