# Supplementary material for: Dasatinib as a treatment for Duchenne muscular dystrophy
Source: Hum Mol Genet. 2015 Nov 24;25(2):266–74. doi: 10.1093/hmg/ddv469 (PMC4706114; doi:10.1093/hmg/ddv469)
Supplement: Supplementary Data [file supp_25_2_266__index.html]

Dasatinib as a treatment for Duchenne muscular dystrophy — Dasatinib as a treatment for Duchenne muscular dystrophy — Supplementary Data 

# Dasatinib as a treatment for Duchenne muscular dystrophy

## Supplementary Data

Supplementary Data

- Supplementary Data - Pdf file
