# Supplementary material for: Dasatinib as a treatment for Duchenne muscular dystrophy
Source: Hum Mol Genet. 2015 Nov 24;25(2):266–74. doi: 10.1093/hmg/ddv469 (PMC4706114; doi:10.1093/hmg/ddv469)
Supplement: Supplementary Data [file supp_ddv469_ddv469supp.pdf]

Supplementary Figures

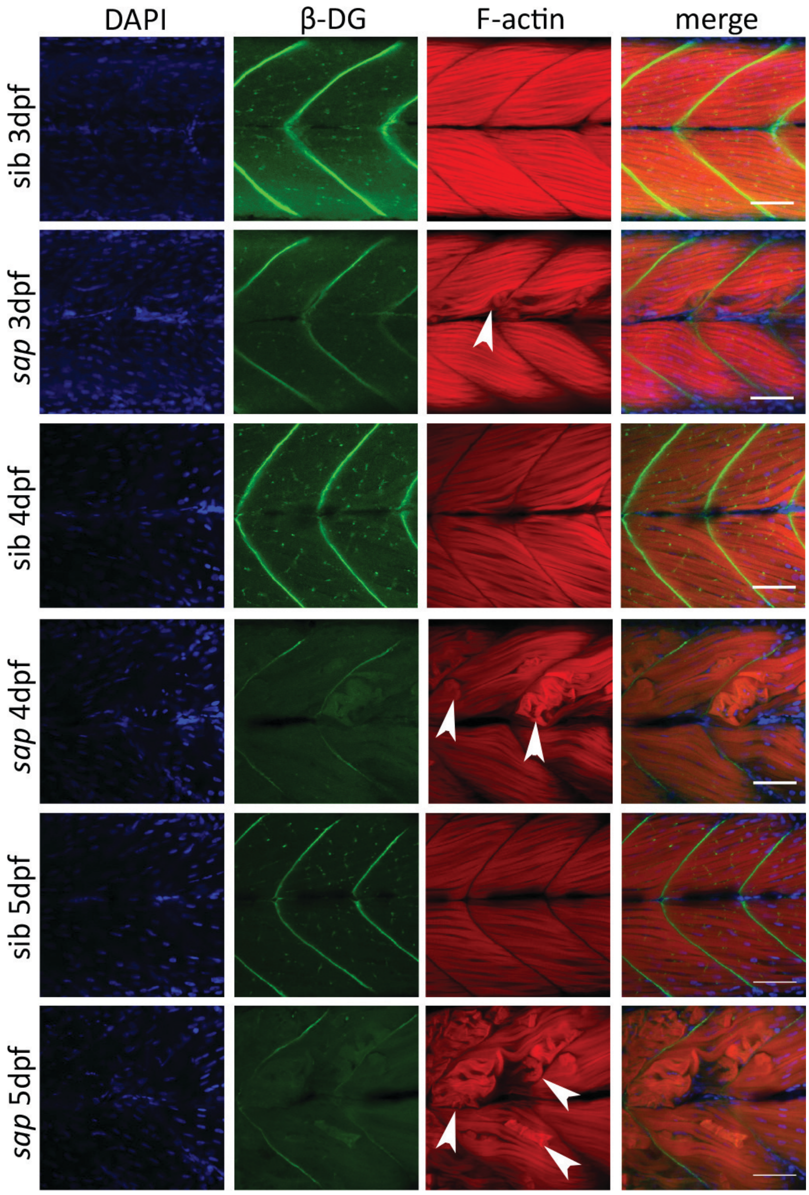

Figure S1. Confocal images of the musculature of 3, 4 and 5dpf *sapje* and wildtype sibling larvae. *Sapje* (*sap*) wildtype sibling (sib) larvae were fixed and stained for nuclei (DAPI, blue) β-dystroglycan (β-DG, MANDAG2, green) and filamentous actin (F-actin, Rhodamine phalloidin, red). Dystroglycan is localised to the vertical myosepta which reduces in intensity in *sapje* larvae, especially at 4 and 5dpf. Rhodamine phalloidin staining shows the increasingly disrupted muscle structure from 3dpf to 5 dpf in *sapje* somites (white arrowheads), compared with the neatly organised array of muscle fibres in sibling larvae (scale bar 50µm).

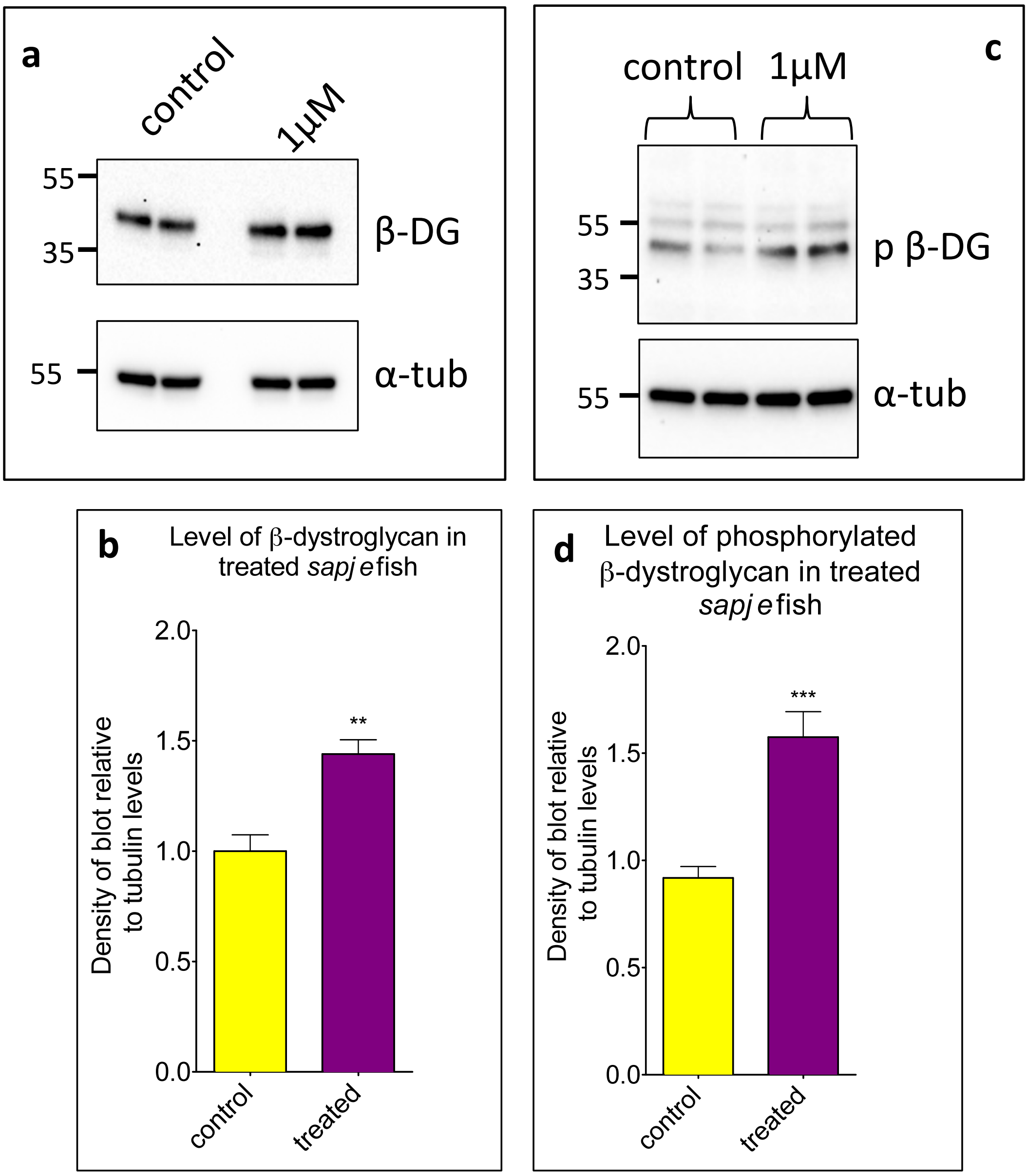

Figure S2, Effect of PYR41 treatment on levels of phosphorylated and non-phosphorylated β-dystroglycan. Lysates were made from embryos treated with 1µM PYR-41 or DMSO only. (A) shows western blots probed against antibodies for β-dystroglycan (β-DG) and α-tubulin (α-tub). (B) The density of the blot probed against β-DG was quantified relative to α-tubulin levels in each sample, and normalised to average control signal. There was a significant increase in the level of β-dystroglycan in larvae treated with PYR-41, compared with controls (Unpaired t-test: t=4.479, df=10, p=0.0012). (C) shows western blots probed against antibodies for phosphorylated β-dystroglycan (pβ-DG) and α-tubulin (α-tub). (D) The density of the blot probed against p β-DG was quantified relative to α-tubulin levels in each sample, and normalised to average control signal. There was a significant increase in the levels of pβ-DG in larvae treated with PYR-41, compared with DMSO only treated controls (unpaired t-test: t=5.063, df=12, p=0.0003). Graphs represent the mean of 6 samples from 3 independent experiments, error bars are SEM.

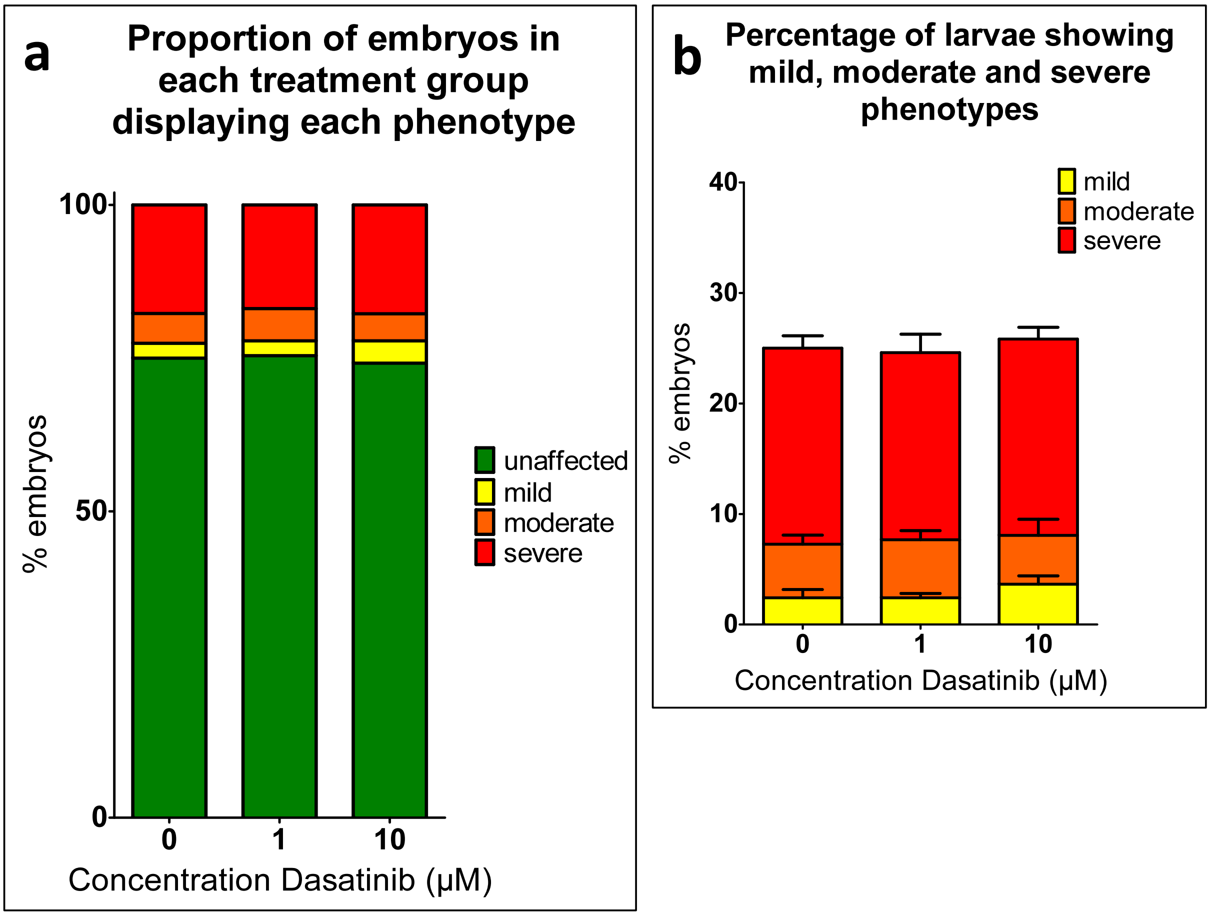

Figure S3 Effect of 48 hour Dasatinib treatment on *dag1* muscle phenotype

Embryos were treated with Dasatinib or DMSO only for 48 hours before a birefringence assay was carried out. The number of larvae showing normal or disrupted muscle birefringence were counted. Those showing disrupted birefringence were further subdivided into mild (disrupted birefringence affecting 1-5 somitic muscle blocks), moderate (6-10) and severe (10+) phenotypes. (a) shows the proportion of embryos displaying each phenotype. (b) shows the proportion of larvae showing mild, moderate and severe muscle damage. There are no significant differences between the percentages of each phenotype in each treatment group. (One-way ANOVAs, mild: F=1.649, df=2,12, p=0.3454, moderate: F=0.1486, df=2,12, p=0.8635, severe: F=0.1277, df=2,12, p=0.8813). Data points represent mean values of 5 independent experiments, with a total of approximately 250 embryos per treatment group. Error bars represent SEM.

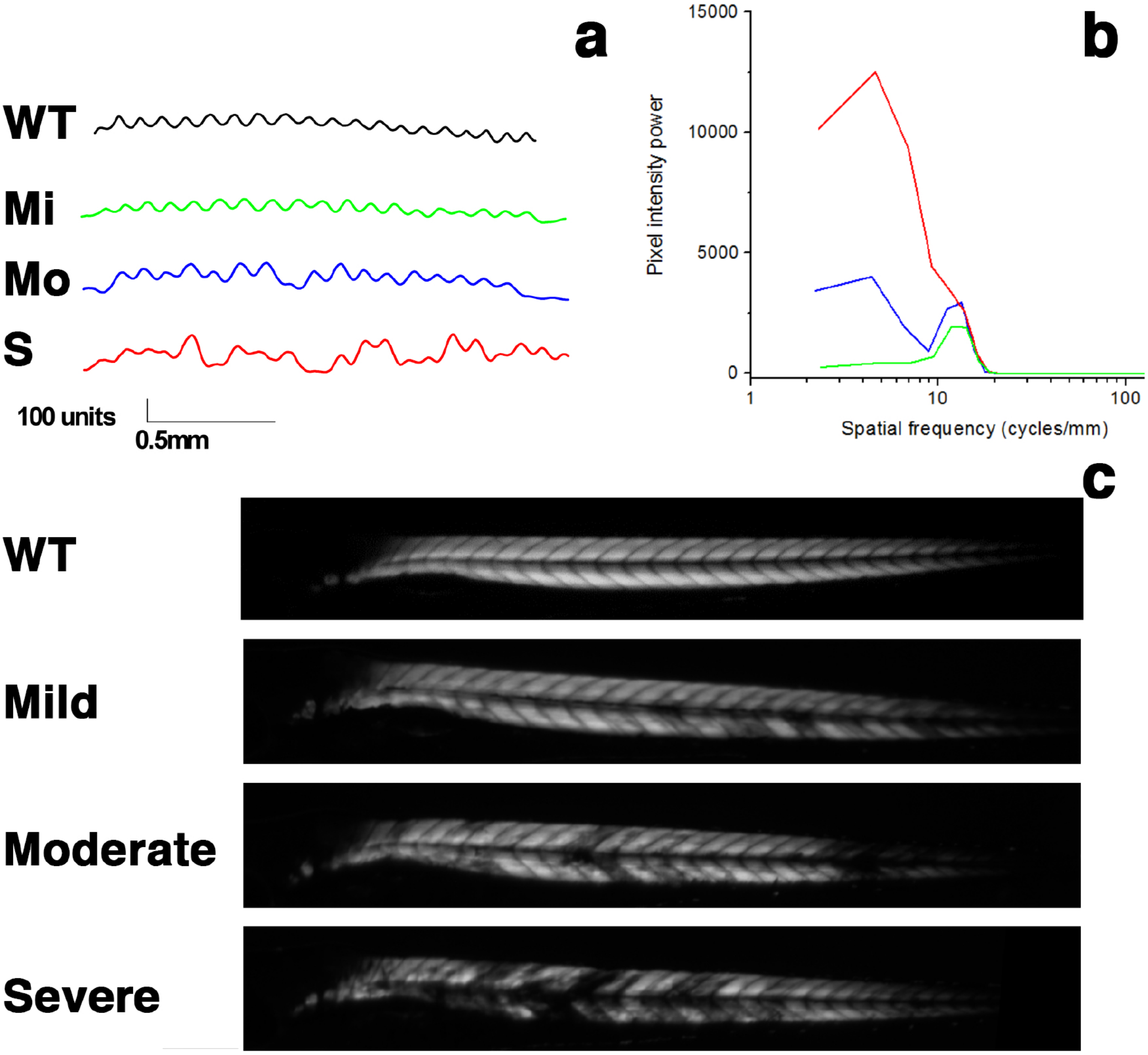

Figure S4. Phenotypic analysis of sapje zebrafish larvae. Panel a shows line scans of normal sibling (WT) and the mild (Mi), Moderate (Mo) and Severely (S) affected sapje muscle birefringence images seen in c. Graph b shows the Fourier transforms of the line scan data for the mild, moderate an severely affected fish in corresponding colours. Fourier transforms of normal sibling fish have a single frequency of just over 10 cycles/mm (not shown), representing the number of somites per mm. Fourier transforms of sapje fish line scans, show an underlying frequency at about 10 cycles/mm, but also exhibit other smaller frequency modes, due to the disruption in birefringence. c shows the images used to create the line scans in and Fourier transforms in a and b and are also representative of the qualitative descriptors 'mild', 'moderate' and 'severe' corresponding to disrupted birefringence across 1"5, 5"10 or 10+ somites respectively. Phenotypic measurements of muscle pathology are based on group analysis of clutches of sapje zebrafish at a given time point, and do not relate specifically to the rate of myofiber breakdown with time.
